# Shaping and shifting schemas on supervised injectable opioid treatment: findings from a cross-sectional qualitative study in two German treatment facilities

**DOI:** 10.1186/s13722-024-00475-5

**Published:** 2024-05-27

**Authors:** Zoe Friedmann, Hans-Tilmann Kinkel, Claudia Kühner, Andreas Zsolnai, Annette Binder, Inge Mick

**Affiliations:** 1 Charité Universitätsmedizin Berlin (Medical University Hospital Charité Berlin), Charitéplatz 1, 10117 Berlin, Germany; 2 Praxiskombinat Neubau, Schwerpunktpraxis für Suchtmedizin (outpatient clinic for addiction medicine), Ruschestraße 103, 10365 Berlin, Germany; 3 Schwerpunktpraxis für Suchtmedizin Stuttgart (outpatient clinic for addiction medicine), Kriegsbergstraße 40, 70174 Stuttgart, Germany; 4 Universitätsklinikum Tuebingen, Sektion Suchtmedizin und Suchtforschung (addiction medicine and addiction research department, Medical University Hospital Tuebingen, University of Tuebingen), Calwerstraße 14, 72076 Tuebingen, Germany; 5 Department of Psychiatry and Psychotherapy, Charité Universitätsmedizin Berlin (Medical University Hospital Charité Berlin), Charitéplatz 1, 10117 Berlin, Germany

**Keywords:** Supervised injectable opioid treatment, Substitution therapy, Patient perspective, Schema theory, Experiential knowledge

## Abstract

**Background:**

Supervised injectable opioid treatment (SIOT) is a promising alternative for people living with opioid use disorder (OUD) who have not sufficiently benefitted from oral opioid substitution treatment. Yet, SIOT utilization remains limited in Germany. We propose that this is due to beliefs, or schemas, on SIOT among people living with OUD. Drawing from medical sociology and social psychology, this study explores the emergence and evolution of such schemas on SIOT.

**Methods:**

We conducted semi-structured interviews with 34 individuals currently in or eligible for SIOT in two German outpatient treatment facilities and paralleled an inductive qualitative content analysis with the exploration of individual cases.

**Results:**

The analysis revealed that peer-to-peer interaction and individuals’ practical experiences in therapy are crucial in constructing and changing idiosyncratic and shared schemas of SIOT. When facing ambiguous information, cognitive strategies like subtyping served to mitigate uncertainty.

**Conclusion:**

This research has important practical implications for integrating experiential knowledge into clinical care and improve information sharing among people living with OUD. A nuanced understanding of the complex network of informal advice-seeking and -giving among people living with OUD is indispensable to adequately expand treatment modalities of proven effectiveness.

**Supplementary Information:**

The online version contains supplementary material available at 10.1186/s13722-024-00475-5.

## Introduction

Opioid Agonist Therapy (OAT) remains the most effective intervention for individuals living with opioid use disorder (OUD) [1, 2]. Yet not all people satisfyingly respond to conventional forms of OAT with oral methadone, morphine, or buprenorphine [3]. Supervised Injectable Opioid Treatment (SIOT) is a promising alternative for people living with OUD who have not sufficiently benefitted from oral OAT. Individuals previously considered treatment-refractory have been shown to improve their health status and social functioning, increase treatment retention and reduce criminal activities and street heroin use when in SIOT [4].

Patients in SIOT inject their substitute (diamorphine, DAM, or hydromorphone) in specialized outpatient clinics under the supervision of healthcare staff. Unlike other forms of OAT, including injectable formulations like extended-release buprenorphine, bolus injections of DAM in SIOT offer a temporary surge rather than sustained stability of mu-receptor agonism. So far, the therapy has been implemented in Canada and several European countries for individuals with severe OUD who fulfil certain eligibility criteria [5]. To be eligible for SIOT in Germany, individuals must be 23 years or older, have used opioids for at least five years, present ongoing intravenous use, have previously attempted treatment for OUD twice, one of which no less than 6 months in oral OAT and have additional health impairments due to continued drug use [6]. Although DAM-based SIOT has been available in Germany since 2009, the estimated need of SIOT is currently not met in Germany. In total, there are only 14 SIOT-clinics in 7 of the 16 federal states in Germany. Further perpetuating this access gap, there is no standardized path to recruit individuals into treatment and relatively few eligible people choose to initiate SIOT [7, 8]. We propose that the underutilization can in part be explained by SIOT’s cultural schema, or how the therapy is understood among people living with OUD.

It has been shown that beliefs about a given substitute are highly influential in patients’ decisions to initiate and maintain OAT [9, 10]. For instance, stigma and misinformation on OAT often lead to the underutilization of effective treatment modalities [11, 12]. Research has predominantly focused on the prevalence and effects of (mis-)beliefs on OAT. The limited evidence on the formation of beliefs about OAT suggests that general societal views, first-hand experiences and a treatment’s perceived effects on peers are important sources of attitudes on OAT among people living with OUD [13, 14]. In this regard, Gryczynski et al. (15:289) describe a highly influential “street narrative […] emerging from a mix of personal experiences and second-hand lore” which is more important than service delivery structure or scientific evidence for engaging people living with OUD in care. Goldsmith et al. [16] explain the importance of information obtained from peers with the heavy reliance of people living with OUD on “street news” about heroin and drug effects before entering OAT. They suggest that even when engaged in treatment, individuals give greater importance to their peers’ accounts than professionals’ knowledge. Similarly, Hunt et al. (17:1753) highlight the significance of methadone’s “street image”, which is “likely to be influenced by the norms and values of the addict subculture”. Yet, there is limited research on how “street images” on OAT are constructed or transmitted and how people living with OUD react to the information they obtain from their peers. Furthermore, despite the substantive scholarship demonstrating the significance of stigma on OAT, little is known on how to change stigmatizing beliefs [12].

With the aim to fill this gap in the literature and better understand the underutilization of effective treatment modalities like SIOT, we translate insights from medical sociology and social psychology to the context of OAT. Specifically, we conceptualize SIOT’s “street image” among people living with OUD as its cultural schema- the therapy’s socially shared representation governing how it is utilized [18]. To investigate dynamics of peer-to-peer knowledge transfer, we add the concept of experiential knowledge, or truth derived from personal experience [19]. In this regard, we understand peer- or social groups as affiliative networks of people who have a comparable status and experience similar situations. In the following sections, we will first introduce our theoretical framework and then turn to the empirical part of this article.

## Conceptual framework

### Schemas

On a basic level, schemas are learned and deeply internalized explanatory frameworks which mediate how persons understand and act upon past and current experiences [20]. Schemas are mainly deployed through automatic cognition and thus lead to fast, intuitive interpretations occurring with little conscious awareness [21]. There are idiosyncratic schemas unique to an individual and cultural schemas, which are intersubjectively shared between members of a social group [22]. Cultural schemas emerge through individual first-hand experience and social interaction, which can be direct (e.g., face-to-face conversation or observation) or mediated (e.g., written accounts) [23]. Once established, cultural schemas are prone to interpersonal reconstruction, which is influenced by group members’ experiences, conditions of the social contact and contingent action. As such, schemas that initially emerged from social interaction can later be reinterpreted by individuals considering their own practical experiences [24, 25]. Conversely, pre-existing schemas and experiences guide whether individuals seek and how they are affected by social interaction [26]. This leads to mutually influencing first-hand experiences, schemas, social interaction and contingent behaviors. According to Strauss and Quinn [23], schemas are thus “flexibly adaptive” rather than fixed.

Despite agreements on their flexible nature, it remains contested how and under which circumstances schemas change. Generally, social interaction and individual experience may either disconfirm or corroborate existing schemas [27, 28]. On a group level, the potential of social contact to change stigmatizing beliefs, a type of cultural schemas [29], is well-documented [30]. Yet, research suggests that in general, cultural schemas are durable in individuals and through time [31]. Even when confronted with contradictory evidence, schemas tend to persevere due to several cognitive and interpersonal processes working against change [27, 32]. For instance, pre-existing schemas influence the uptake of new information and often lead to schema-consistent interpretation and re-transmission [33, 34].

Distinctions between schemas vs. non-schemas are often gradual rather than definitive [35:536]. Despite conceptual inconsistencies, schemas are useful explanatory frameworks in multiple disciplines in and beyond sociology [36]. Some scholars describe associative neural networks making up cultural schemas on an algorithmic level. Building on insights from this approach, we analyze schemas on a “functional level” [21] to explore the construction and evolution of schemas on SIOT among people living with OUD. As scholarship on cultural schemas has often focused on social macro-processes, it is useful to also consider work on the practical co-construction and transfer of knowledge in subcultures, which we turn to now.

### Experiential knowledge

Our theoretical framework also includes the concept of experiential knowledge, which has gained much attention in the past decades in healthcare [37] and beyond [38, 39]. In contrast to established occupations ’ systematically accumulated professional knowledge, experiential knowledge is derived from personal experience [19]. This includes vicarious experiential knowledge gained from talking with and observing others dealing with a particular situation [40]. Abel and Browner [41], for instance, distinguish “embodied” knowledge obtained through one’s own, direct bodily experience and “empathetic” knowledge gained through close emotional ties with those experiencing a phenomenon. Upon individual reflection and organization, initially implicit experiences are transformed into re-countable experiential knowledge. Individuals’ experiential knowledge is fluent across time and context [42], which leads Boardman [43] to differentiate between cumulative experiential knowledge shaped by linear, “quasi-paradigmatic shifts” and fragmented experiential knowledge, where “different, but equally accurate” truths can coexist.

Despite their differences, experiential and professional knowledge are neither inherently conflicting nor mutually exclusive. Rather, they can complement and co-shape one another both on an individual and group level [44–46]. However, frictions arise when discordant information is presented about the same phenomena (19:448). In the medical context, power differences inherent in the doctor-patient relationship [47] suggest that professional knowledge trumps experimental knowledge when tensions arise. Yet, resistance to professional in favor of experiential knowledge is highly context-specific and rather gradual than definitive [48, 49], particularly in the context of OST [50].

According to Noorani [51:65], experiential knowledge relies on “collective meaning-making” between members of a peer group. Examples of such groups include Alcoholics Anonymous, mutual aid groups for single parents or for people who stutter and patient organizations of people living with fibromyalgia or chronic fatigue syndrome [37]. In the process of “collective meaning making”, individuals share their experiential knowledge and subject it to interpersonal evaluation, whereby it obtains validity through its embeddedness in real-life experience and assumed representativeness for those who find themselves in similar situations [52]. Sharing and validating individuals’ experiences is facilitated by “the feeling of being understood as well as the open, natural communication between those with similar experiences” (19:450). Eventually, this leads to the production of a collectivized pool of knowledge. Particularly for potentially stigmatized and vulnerable groups, pools of experiential knowledge can be important resources to mediate decision-making processes in uncertain situations [53, 54]. Yet, as Halloy et al. [37] point out, the transfer and uptake of experiential knowledge is highly context specific, cognitively challenging and a matter of degree- regardless of how similar individuals’ situations might be. Mazanderani et al. [55], for instance, provide a rich account of the “identity work” necessary to transfer others’ experiences onto one’s individual situation and highlight the plurality of experiential knowledge of people living with the same diagnosis. While the co-production and utilization of experiential knowledge can occur in a variety of mediation spaces, prior research has focused on organized forms of collective meaning-making, e.g., in self-help groups or internet fora [37]. Little attention has been paid to informal networks as described among people living with OUD.

## Methods

Spoken discourse can be understood as “the external matrix of all deeply internalized cultural schemas” (18:230). Although schemas and their effects are seldomly formulated as explicit declarative propositions [20], they can thus be reconstructed through linguistic data [56]. This makes interviews a suitable method to explore individuals’ schemas and the dynamics underlying their formation.

This article builds on data from a qualitative interview study with people currently in or eligible for SIOT. Specifically, we investigated the patient perspective on barriers and enabling factors for SIOT-initiation and -maintenance and on how to improve the therapy [10, 57]. Inclusion criteria for the interview study were purposefully broad to include different experiences with and perspectives on SIOT. All people currently receiving OAT who fulfilled German eligibility criteria for SIOT [6], had an adequate level of English or German and were able to provide informed consent were eligible. Our research was approved by the ethics committee of Landesärztekammer Baden-Württemberg (AZ: F-2022-002). We followed the *consolidated criteria for reporting qualitative studies* [58] and provide further information regarding our methods in supplementary material 1.

To develop our semi-structured interview guides, authors 1, 2 and 3 first screened the existing literature on the initiation and maintenance of OAT and barriers to care to identify common themes, key issues, and potential areas of interest related to our research objectives. The specific interview questions were additionally informed by relevant qualitative research exploring individuals’ experiences with both oral and injectable OAT and the extensive clinical experience in OUD-care of several members of the research team. Prior to data collection, the interview guides and the study’s purpose were discussed in a focus group including people living with OUD and persons providing psychosocial support in the Berlin clinic. This was to ensure that the study design was feasible, non-judgmental and of relevance for people with lived experiences [59]. As the feedback obtained during the focus group session was affirmatory, it did not result in significant changes.

### Participants

We recruited participants from two German outpatient SIOT-clinics. One clinic, located in Stuttgart, is the sole provider of SIOT in the German federal state of Baden-Württemberg. It accommodates 260 patients in oral OAT and 140 in SIOT. The other clinic, situated in Berlin where two SIOT-clinics exist, has 220 patients in oral OAT and 103 in SIOT. While most patients reside in Stuttgart and Berlin, some also travel from adjacent areas to access SIOT. Both clinics allow for interaction and knowledge transfer among patients in oral OAT and SIOT, e.g., in the clinics’ surroundings. Stuttgart, situated in the south of Germany, has a population of approximately 650,000 inhabitants. In contrast, Berlin, located in the east of Germany, has around 3.8 million inhabitants.

All patients of the respective clinics were screened for eligibility and grouped into currently being in SIOT or currently being in oral OAT. For people in oral treatment, we differentiated between individuals ever and never having been in SIOT. Stratified by age and self-assigned gender to reflect the study clinics’ patient population, eligible people were randomly selected from the respective groups. Selected individuals were offered to participate in the study by the clinics’ staff and provided with a study information and consent form.

Guided by previous experience from members of the research team, the consultation of outside qualitative researchers during the planning phase of the study and the concept of information power [60], we set an initial number of participants to 16 individuals currently in SIOT and 12 currently in oral OAT. These numbers evolved throughout data gathering and preliminary analyses. For instance, we did not expect patients’ enthusiasm to participate and initially over-sampled people in SIOT. Yet, all sampled individuals wanted to participate, and we continued to schedule interviews until no new topics kept emerging. Conversely, we were not able to recruit 12 individuals in oral OAT eligible for SIOT. Finally, 23 participants currently in SIOT and 11 participants currently in oral treatment, of which 4 had ever received SIOT, were included in the study.

### Data collection

One-on-one semi-structured interviews were conducted by author 1, a female medical student trained in qualitative research who was unknown to participants prior to data collection and not providing care at the study sites. The interviews were held between May and August of 2022 in private rooms in the respective clinics. Prior to all interviews, author 1 again explained the study’s purpose, her position and confidentiality protocols. It was stressed that participation was voluntary and the decision to take part unrelated to participants’ care at the clinic. Upon informed written and verbal consent, the interviews were audio-recorded and supplemented with field notes (e.g., on participants’ non-verbal cues, contextual information and author 1’s personal reflections).

Interviews ranged from 18 to 61 min. All interviews started with an open question inviting participants to reflect upon their initiation of SIOT and/or oral OAT. Participants who had ever been in SIOT were asked about their experiences in treatment and thoughts on discontinuing treatment. Depending on their individual circumstances, participants currently in oral OAT were asked if and why they would or would not (re)enroll in SIOT. All participants were asked if they had any suggestions to improve SIOT and encouraged to share any further thoughts upon ending the interview. While the questions were broad and invited participants to lead the course of the interviews, author 1 used follow up and probing questions when deemed appropriate. After the interviews, participants were invited to contact author 1 for any further inquiries or having transcripts of their interviews returned to them. All participants (including those consulted prior to data collection and publication) were given a compensation of 20€, which was self-financed by the research team. We took measures to mitigate potential conflicts of interest arising from this arrangement (e.g., repeated critical reflections and consulting Charité’s *Office for Research Integrity*).

### Data analysis

All data were analyzed in German. The illustrative quotations for this article were translated from German to English in forwards-backwards technique by bilingual academics (IS, GS) for concurrent validity [61]. Assisted by NVivo transcription-software, pseudonymized interviews were transcribed verbatim and identifying information was removed. For systematic coding, transcripts and fieldnotes were imported into MaxQDA (2022) software. We included data from all participant interviews in the analysis, which was informed by qualitative content analysis in a structured-thematic approach [62, 63] and commenced after completing the first 4 interviews. Following repeated close reading of the transcripts, author 1 inductively derived initial major categories (e.g., perceptions of SIOT). A paper describing the content of different perceptions on SIOT and how these influence therapy-initiation has been published elsewhere [10].

**Fig. 1:**
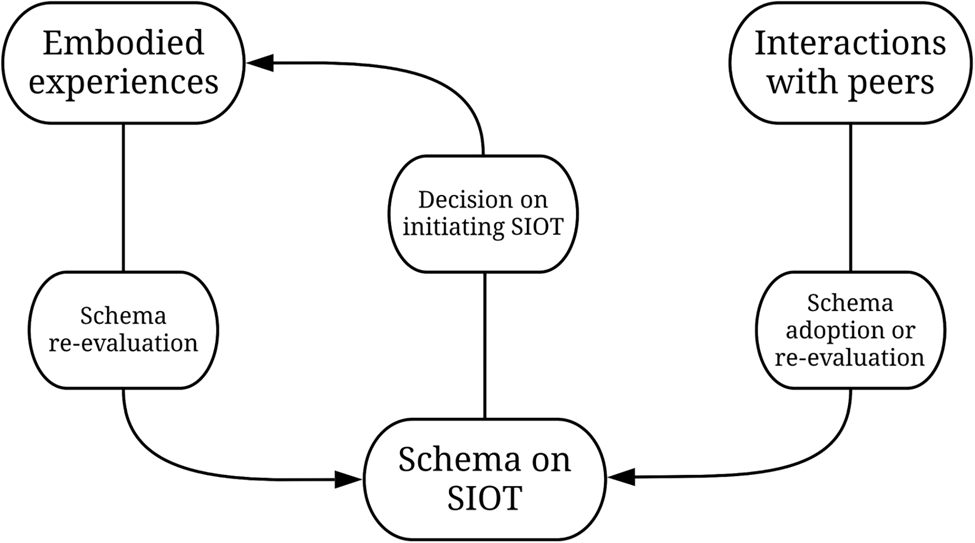
Interaction and embodied experiences shaping schemas on SIOT. SIOT: Supervised Injectable Opioid Treatment

In an iterative, cyclical approach supplemented by reflexive memo-writing, author 1 derived increasingly differentiated sub-categories (e.g., factors influencing perceptions of SIOT, differentiated in e.g., embodied experiences and interactions with peers). The emerging category system was refined during independent coding by author 1 and the research supporter, where discrepancies were discussed until consensus was reached. The final category system guiding the analyses presented in this paper was applied independently to about 20% of the material and can be found in supplementary material 1. During qualitative content analysis, author 1 noticed overlapping elements with work from the social sciences. Alternating between theory and our empirical material, we consequently developed our theoretical framework. This allowed us to better understand dynamics and relationships between our categories and surpass the descriptive stage of qualitative content analysis. Paralleling cross-case analyses, author 1 reviewed schemas on SIOT in individual cases to compare emerging dynamics across interviews with those of individual accounts and identify disconfirming cases. Thus, the model guiding the interpretations of our results (Fig. 1) emerged as a synthesis of individual and cross-case analyses and was present in all cases in different specificities.

To facilitate critical reflexivity, author 1 kept a log of all methodological and analytical steps made during the research process. In regular verification meetings, author 1 discussed the category system and analytical approaches with variable members of the research team. During these meetings, researchers providing care to study participants only reviewed de-identified illustrative quotes. We continuously reflected upon the appropriateness of our theoretical framework and presented our analytical approaches at interdisciplinary interpretation groups and colloquia for intersubjective validation. We acknowledge that the data we gathered in interviews were co-created by the researchers and participants [64]. Prior to publication, we individually discussed this article’s summary results with 3 people living with OUD. They affirmed that the interpretations we made aligned with their informed lived experiences.

## Results

**Table 1:** Aggregated data on participant characteristics. SIOT: Supervised Injectable Opioid Treatment

Participant characteristics	Number of participants	Total
Currently in SIOT	23	34
Currently in oral treatment, never having been in SIOT	7	
Currently in oral treatment, previously having been in SIOT	4	
Male	22	34
Female	12	
From Stuttgart	18	34
From Berlin	16	
Age: 31 to 59 (mean 43) years		

*Note* The sample included people of color and non-German citizens. To protect the identity of these participants, we do not disclose additional information on diversity metrics.

Most participants’ schemas of SIOT emerged from the information they derived from direct interactions with peers and their own experiences in treatment, as shown in Fig. 1. Less frequently mentioned sources of knowledge were mediated accounts of experiential knowledge such as SIOT-clients’ testimonials in magazines or online. When participants obtained knowledge about SIOT from medical professionals or social workers, they often described corroborating this by observing and talking to their peers. Rather than shaping schemas on SIOT, professionals were depicted as managing the treatment of co-morbidities, practical gatekeepers and a source of psychosocial support. The importance given to information gathered through interactions with peers stemmed from a feeling of mutual understanding, facing similar problems, previous and/or ongoing negative experiences with healthcare professionals and the ubiquity of informal exchanges among peers in- and outside of the clinic.

In the following sections, we explore how interactions with peers and embodied experiences in treatment influenced participants’ schemas of SIOT. As we describe dynamics between categories, the presentation of our results does not follow the structure of our initial category system. We will first outline accounts in which participants entirely changed their understanding of SIOT considering information that conflicted with their prior schema. We then turn to more ambiguous situations where conflicting views coexist. While some participants comfortably accommodated partly contradicting information in their schemas, others expressed uncertainty arising from this. Several participants developed cognitive strategies to make sense of conflicting information by isolating individual accounts from one another or devalued their peers’ credibility. We end this section with participants’ practical suggestions for accurate and adequate information transfer. Participants whose anonymized data are presented below were assigned gender appropriate pseudonyms and selected because they most clearly capture our key findings.

### Consistent schematic changes

Several participants described profound schematic changes resulting from the embodied experiences they made in treatment. Angela, a female in her 40s, has been in SIOT for 2 years after a long period of uncertainty on enrolling. Before SIOT, she has been on and off oral OAT for over 10 years, where -like most participants- she has never been able to control her co-use of street drugs. In SIOT, she feels satisfied and that her life has become more stable. In her case, information from peers influenced her initial schema of SIOT, which she then questioned considering conflicting empathetic experiential knowledge. Finally, her embodied experience invalidated and replaced her initial schema.

“You have to have x therapies, nothing can help you anymore, you have to be totally f*cked up to even get into the [SIOT] program”. Rumors like that were going around and that discourages you from entering the program. And actually, it’s not like that now at all. If you look at some of the people upstairs in our area, you wouldn’t think that they are in the program. They go to work normally and everything. Yes, but that scares people on the scene. Because they have totally different ideas if they don’t know someone [in SIOT] themselves. And also, when you say, “the program”- and I was also like “no way I’m going, I would never go in”. And because [boyfriend] was in there and another mate, that’s when you start- I also saw how [boyfriend], how it did him good and everything. And then I just went along.

While social interaction profoundly shaped Angela’s schema, this was not as clear for other participants. Nadine, a female in her 40s, has been in SIOT for 8 months after being unstably treated in oral OAT for nearly 20 years. She radically changed her schema of SIOT following positive embodied experiences in treatment. Although Nadine had several close friends in SIOT who told her that they were greatly benefitting from the treatment and encouraged her to initiate SIOT as well, she actively blocked these influences until she felt like there was no other way to control her co-use. She explains:

I’m really a patient who goes from “no way” to “10 stars”. And I wouldn’t have thought that myself. Really, honestly. Thank God [my friend] convinced me. So, I often think to myself “Yes, I should have done that much earlier”. Because even back then [my friends] were saying to me, “Come on Nadine, you should go into the diamorphine program, you always smuggle your sh*t out here [to inject your oral substitute]”. And I couldn’t help it, I was always like “Whoa, now stop doing this, for real!” and stuff like that. And then I always played tricks with the masks and ah, really stupid. I was like, “No, I never want to do that, get lost!”

Nadine could not clearly depict the source of her initial schema. When inquired on why she held a negative image on SIOT, she responded:

I just don’t know; I think that was really pure ignorance […]. That came from me. Like, that was my attitude.

As many participants whose embodied experiences paradigmatically changed their schema on SIOT, Nadine even reversed her image of methadone treatment. She now juxtapositions SIOT-patients (including herself) with individuals in methadone maintenance and says that

I still know and see far too many people in the methadone program. That sh*t should actually be scrapped. […] I just think that the clinics that offer methadone should think about whether [providing SIOT] might not make more sense.

### Integrating conflicting information and avoiding uncertainty

Another group of interviewees did not report such radical schematic shifts when confronted with new information. These participants often had to accommodate conflicting, but coexisting information from interactions and embodied experiences. Uwe, a male in his 50s who started oral OAT in the 1980s, was in SIOT for about a year and liked many aspects of the treatment. He went back to oral OAT after “turning blue” and having to be oxygenated twice in SIOT. Notwithstanding his negative embodied experiences, he maintained a positive view of SIOT.

The substitute itself, that’s good. But I wanted to- I’m a bit fond of it, even if it’s not as great as it could be. But I don’t want to die because of it. And that’s why I left the program.

Not all participants could easily integrate conflicting information. Stefan, a male in his 40s, started SIOT because other patients conveyed a positive schema of the therapy. However, he felt uncomfortable in SIOT and decided to go back on oral OAT after a month. Despite expressing a consistently negative opinion towards SIOT, he had trouble to integrate his embodied experience with the information he obtained from peers.

I don’t know many dropouts. I don’t really know anyone, except myself. So, I don’t know what they all find so great about it. […] I can’t see anything good about it. For me, it was all just bad.

This trouble was also evident in the accounts of many interviewees eligible for but never having been in SIOT. Unable to construct a consistent schema on SIOT, they faced uneasiness in their decision-making processes regarding the therapy. Katrin, a female in her 30s in oral OAT who feels burdened by her co-use of cocaine and alcohol, repeatedly expressed insecurity arising from the information she obtains from her peers.

I have seen or- or know many people who take [DAM] and really only the very smallest percentage is fully satisfied with it, and a few do really well with it […]. But like I said, the ones who maybe find it positive, you don’t see them anymore. So, I don’t know what to think about it. In the end, it would have to help me.

Several participants developed strategies to avoid uncertainty and discomfort when facing conflicting information. Some individualized and subtyped the input they got and refrained from general inferences. Bettina, a female in her 30s currently in oral OAT whose brother discontinued SIOT, said that

many aren’t on the streets anymore. They all take diamorphine and are happy with it. Many say “It’s good for us. I’m satisfied. So, I try to go about my regular day that way”. It varies. You can’t put everything on one person […]. But I just say [SIOT] is not for me. It’s much too strong, I saw that with my brother. That’s what made me scared of it.

Additionally, interviewees frequently individualized and contrasted their embodied experiences with those of their peers to maintain consistency. Thomas, a male in his 40s who has been in SIOT for 4 years, stated that

I do great with it. There are some people who also do great with it, but others don’t do so well. So, it’s different for- it’s different for each person with diamorphine.

Another group of participants dealt with conflicting information by devaluating their peers’ accounts. Sascha, a male in his 40s, initiated SIOT to avoid a prison sentence despite the negative view on the therapy he adapted from his peers. The positive embodied experiences he made in treatment changed not only his schema on SIOT but also how he evaluates what he heard from his peers.

Now, looking back on it, if I’d known that before, I probably would have come sooner. It’s just that I got misinformation from other people about [SIOT]. […] But those people aren’t even in diamorphine and they already have preconceptions against it. And then they say something like that about it.

### Participants’ suggestions for improvement

While most participants depicted interactions with peers as their primary source of knowledge, they also problematized misinformation circulating among people living with OUD and suggested educational interventions to combat this. Participants advocated for including people with lived experiences and greater interpersonal exchange between patients, medical personnel, social workers and individuals currently not engaged in treatment. Sebastian, a male in his 30s who has been in SIOT for the past 7 years, explains:

If I had had someone at [harm reduction NGO] or something who were using themselves, who could tell me about it first-hand or from whom I could pick up tips, where I knew “hey, they’ve got the sh- They were also on the street once, they’ve been injecting long enough themselves”. Maybe I would have picked up one or two tips from them.

Practical suggestions to improve interpersonal exchange regarded “patient advocates” in the clinic to mediate between medical personnel and patients, patient-led group sessions and inviting people into the clinic to get their own impression on SIOT. Julia, a female in her 30s who has been in SIOT for one year, suggested

that we are asked more often. I mean, we are the ones who have to go there and need it. And yes, I think it would also be nice to publicly […]. People should be shown what’s happening here and what kind of people are coming here.

## Discussion

Building on work from medical sociology and social psychology, this study investigates the. social construction of schemas on SIOT in two German outpatient OAT-clinics. We find that peer-to-peer interaction and embodied experiences are the predominant influences on participants’ schemas regarding SIOT. This is in line with prior work from the social sciences [21] and addiction medicine [15]. Focusing on the context of OUD, our findings contribute to the growing literature that examines the production, spread and utility of experiential knowledge.

### The importance of vicarious experiential knowledge in developing schemas

Prior to treatment-initiation, we found vicarious experiential knowledge on SIOT (both second-hand and empathetic) as highly influential in the development of participants’ schemas. This contextualizes recent findings on the relational qualities of treatment engagement in SIOT, where several participants initiated SIOT due to the experiences their partners had made in treatment [65]. Interestingly, work on buprenorphine found that individuals formed their opinion on the medication mainly through embodied experiences after obtaining it illegally on the street and only partly through interactions with peers [15]. This difference is likely caused by the supervised application in SIOT, which makes the diversion of a relevant amount of injectable DAM improbable and restricts first-hand experiences to clinical settings [66]. Such unique nuances in the delivery of different OAT modalities may engender further distinct patterns in the formation of treatment schemas. Thus, more context-specific research is needed to better understand the influence of peer experiences on treatment perceptions in OAT modalities beyond SIOT.

Strikingly, only a minority of participants based their schemas and decisions regarding SIOT on professional forms of knowledge. Generally, participants neither explicitly devalued nor mistrusted professional knowledge, as previous work on OAT [50] and experiential knowledge [37] suggests. The reliance on informal interactions with peers might be a residual habit from before entering and stabilizing in treatment [16]. Another probable explanation are the “different conceptual worlds” [67] patients and medical professionals operate in, which is particularly pronounced in treating OUD [68]. Coupled with the unique feeling of mutual understanding and “natural communication” among peers [19], this might render professional knowledge less relevant. Although beyond the scope of this study, disentangling individuals’ reasons for (not) relying on professional knowledge regarding OAT is an important avenue for future research. Simultaneously, peer-to-peer knowledge transfer must be considered in all efforts to engage individuals in treatment.

### Adapting schemas

Over time, participants described frequent adaptations of their idiosyncratic schemas and situated decision-making processes. Generally, embodied experiences in SIOT that put pre-existing schemas to the test were the most prominent stimuli for change. This mainly regarded changes towards more positive, but also towards mixed or negative schemas, which complements previous quantitative [69] and qualitative [70] findings on patients’ variable satisfaction-trajectories in SIOT. We did not find that individuals who initially held negative schemas on SIOT maintained these in treatment. This goes against studies on methadone, where patients kept negative attitudes toward the substance in therapy even when they thought that methadone has had a positive effect on their lives [17, 71].

Additionally, participants adapted their schemas considering social interactions with peers, which is in line with prior research [30]. In this process, they gave varying importance to the information they obtained. Our findings concur with Mazanderani et al. [55] and Borkman [19], who argue that the assumed representativeness and uptake of vicarious experiential knowledge is situationally and temporally contingent. Furthermore, social learning and schema theorists propose that the selective internalization and interpretation of new, potentially ambiguous information serves to construct coherent and eventually durable cultural schemas [23, 72]. In this regard, cognitive mechanisms such as subtyping and individualization [27] were utilized by many participants in our study.

Yet, our findings on incongruent schemas on the individual and group level suggest that factors beyond schema-consistency should be considered. Here, we found that some participants prioritized emotionally demanding and affectively laden interactions, as suggested by Hunzaker [73]. Furthermore, the “friendship potential” and repeated interaction among patients in the same clinic likely mitigated schema-consistent interpretations and allowed for change [26]. We can only speculate about additional influences (e.g., the selective uptake of information due to social hierarchies within treatment and recovery settings [74]). To reach comprehensive conclusions, future studies should systematically evaluate how people living with OUD prioritize the information they obtain from their peers.

### Fragmented experiential knowledge as a resource

Our results disagree with studies on the utility of experiential knowledge in uncertain situations [53]. Rather than serving as a valuable resource, conflicting experiential knowledge and second-hand accounts caused insecurity in decision-making on SIOT, which is more in line with Boardman [75]. This might be because, in our context, we found no organized collective pool of experiential knowledge or consistent cultural schema, but an inconsistent, informally transmitted agglomeration. Boardman [43] argues that experiential knowledge can take paradigmatic shifts or contain fragmented, partially contradictory information. For Boardman [43], however, intraindividual fragmentation characterizes experiential knowledge as a “living” resource. We rather found fragmentation to produce uneasiness for many participants. Considering these differences, further research in the context of OUD is needed to tap the potential of experiential knowledge and peer-to-peer informational exchange and improve SIOT-engagement.

### Practice implications

Our findings on peer-to-peer transmissions of experiential knowledge and the suggestions made by participants have several practical implications. It would be wise to enhance peer-to-peer support and the organized sharing of experiential knowledge in clinical care for OUD [76]. While evidence on peer support is limited and shows only modest impacts [77], this might be due to barriers towards the meaningful implementation of experiential knowledge in healthcare services [78–80]. In particular, tokenism [52], exploitation [81, 82] and the commodification [83] of people with lived experiences must be avoided through careful planning and realization [84]. Enhanced sharing of experiential knowledge among peers is no panacea against the spread of misinformation and distrust [85, 86]. Thus, simultaneous efforts should be made to mitigate problematic beliefs and promote accurate information sharing among staff, patients and patients’ social networks [87, 88]. Social contact interventions, e.g., inviting people into the clinic to get their own impression on SIOT, as some participants suggested, might be a way to reach individuals beyond patients’ direct networks [89] and reduce inner-group stigma [90]. However, one must be wary of potential unintended consequences of these interventions (e.g., perpetuating perceptions of dangerousness or increasing self-stigma) [91]. Thus, while implementing such interventions, careful consideration of both their potential impact and strategies to mitigate any negative outcomes is crucial.

Another consideration regards eligibility criteria for SIOT, which were frequently mentioned as a presumably objective source of stigmatizing schemas. The high costs and elevated rates of adverse events in SIOT necessitate thorough considerations on who to enroll [92]. Nevertheless, regulations on OAT are not isolated from larger socio-political contexts and can reinforce structural stigma [93, 94]. Our findings highlight that regulations must be carefully communicated, frequently re-evaluated and potentially adapted to not enhance prejudice, marginalization and the underutilization of effective treatment modalities.

## Strengths and limitations

A strength of our research is its diverse study population. We went beyond previous work by including people eligible for, previously in and currently in SIOT. Participants had various treatment trajectories and could speak to interpersonal dynamics both in- and outside of therapy. The importance of informal knowledge transfer among peers in out-of-care populations found in this study and previous research suggests that, to some extent, our findings are transferable to OAT modalities beyond SIOT and to individuals currently not engaged in treatment. Furthermore, coupled with the coherence of accounts from both study sites, it is likely that our findings are transferable to geographical regions beyond our study sites. Just like most previous research on OAT, however, we conducted our study in urban areas and cannot necessarily speak to rural regions.

A further important limitation of this study is that we solely relied on cross-sectional interviews. A longitudinal design would have mitigated recall biases and might have allowed us to explore dynamics in individuals’ schemas over time more rigorously. Additional qualitative methods, e.g., participant observation, might have enriched our analyses and shed light, for instance, on the configuration of interpersonal networks underlying the dynamics we investigated [95]. Because we focused on the experiences of people living with severe OUD on the individual and group level, we cannot necessarily speak to processes on higher societal levels (e.g., the public creation of stigma on OAT), which likely interact with what we found on the micro-level [27].

Furthermore, presenting our findings in English introduces some constraints. It is possible that certain nuances inherent in the original German data have been modified or overlooked during the translation process. To mitigate this risk and ensure that the English version remains close to the original German data, we employed forward-backward translation and deliberated on all translation decisions both within and outside the research team. Finally, we integrated people with lived experiences only on a “consult” level due to ethical and practical considerations [59]. While this allowed different stakeholders’ priorities and reflections to be implemented into our study, the main responsibility remained within the research team and social desirability responses might have affected the affirmatory outcome of our consultations with people living with OUD.

## Conclusion

This study explored how schemas on SIOT are built, transmitted and transformed among people living with OUD. Peer-to-peer interactions and embodied experiences were the predominant influences on participants’ schemas regarding SIOT. Thus, effective interventions must be based on an understanding of the complex social dynamics involved in (not) seeking treatment and peer-to-peer knowledge transfer should be acknowledged in all considerations on SIOT. In this regard, the accumulation and sharing of experiential knowledge should be supported. Informal exchanges should be supplemented with organized information sharing and measures to mitigate problematic beliefs that might produce insecurity and harm. In all these efforts, individual preferences and strategies of knowledge-seeking must be respected to meaningfully improve service provision for people living with severe OUD and assist them to achieve their individual treatment goals.

### Electronic supplementary material

Below is the link to the electronic supplementary material.

Supplementary Material 1

## Data Availability

The data that support the findings of this study are not openly available due to reasons of confidentiality. Anonymized data can be made available from the corresponding author upon reasonable request.

## Abbreviations

DAM: Diamorphine
OAT: Opioid Agonist Therapy
OUD: Opioid Use Disorder
SIOT: Supervised injectable opioid treatment
