# Supplementary material for: Shaping and shifting schemas on supervised injectable opioid treatment: findings from a cross-sectional qualitative study in two German treatment facilities
Source: Addict Sci Clin Pract. 2024 May 27;19:45. doi: 10.1186/s13722-024-00475-5 (PMC11129426; doi:10.1186/s13722-024-00475-5)
Supplement: Supplementary file 1 — Supplementary Material 1 [file 13722_2024_475_MOESM1_ESM.docx]

Supplementary material 1: Further information regarding our methods

1. Further information on data collection:

1.1 Participant selection:

Eligible people were grouped into currently being in SIOT or currently being in oral treatment. People currently in oral treatment were additionally grouped into individuals ever having been in SIOT and never having been in SIOT. Stratified by self-assigned gender and age to reflect the respective clinics´ clientele, eligible people were randomly selected and offered to participate in the study face-to-face by the clinics´ nurses, doctors, or psychologists. We acknowledge that random sampling is not a standard in qualitative research. This strategy was nevertheless deemed most appropriate for this study as it reduced bias and increased the heterogeneity of the experiences included. Strategies such as purposive sampling would have introduced biases stemming, for instance, from power differences or inter-personal frictions between clients and staff at the respective clinics. In addition, our strategy reduced self-selection bias. Particularly people living with severe addiction might be deterred from actively responding to an open invitation to participate in a research project, for instance due to past negative experience with healthcare staff and/or researchers.

1.2 Sample Size:

We set an a priori number of participants to 16 people currently in SIOT and 12 people currently in oral treatment, which evolved during data gathering and preliminary analyses. Finally, 23 participants currently in SIOT and 11 participants currently in oral treatment, of which 4 participants had ever received SIOT, were included in the study.

-Planned Number of Participants:

As a rough approximation, we set an a priori number of participants to 16 people currently in SIOT and 12 people currently in oral treatment. This was guided by previous experience from members of the research team, the consultation of outside qualitative researchers during the planning phase of the study, and the concept of information power (Malterud et al., 2016). Furthermore, practical considerations meant that we could not include as many participants in oral treatment who are eligible for SIOT as people who are currently in SIOT. Individuals eligible for but not in SIOT continue to use drugs intravenously and often do not improve significantly in oral treatment. In both study sites, this population was thus quite small and harder to reach compared to people in stable substitution treatment. Because we anticipated that not all sampled individuals would agree to participate in the study and that some might not appear to their scheduled interviews, we initially sampled 25 people currently in SIOT and 15 people in oral treatment and eligible for SIOT to reach our intended number of participants.

-Actual Number of Participants:

During data gathering and supported by preliminary analyses, we continuously evaluated the adequacy of the initially set number of participants. All individuals currently in SIOT we sampled wanted to participate, nearly all appeared to their scheduled interviews, and new topics kept emerging even as we had completed 16 interviews. We thus included 23 participants currently in SIOT into the study instead of the 16 participants we had planned initially. We only included 11 instead of 12 participants in oral treatment because we were not able to recruit more participants, partly because individuals from the small group of eligible people declined to participate and partly due to non-appearances to scheduled interviews.

-Non-participation:

2 individuals (both currently in oral treatment and never having received SIOT) who were sampled declined to participate without giving a reason and 4 participants who had given consent repeatedly missed their scheduled interview and were thus not included in the study. In general, participants appeared to welcome the opportunity to share their experiences. No participants withdrew consent after the interview.

1.3 Interview Setting:

Author 1, who was unknown to participants prior to the interview and not involved in their medical care, conducted all interviews. Prior to all interviews, Author 1 explained the purpose of the study (getting to know more about SIOT and to improve it on the long run), her position (a medical doctoral student unaffiliated with the clinic and not providing care), and confidentiality protocols (storing all identifiable data password-protected with strict access restrictions, de-identifying data upon transcription and sharing only anonymised data). Participants were told how their data would be used in the further research and were offered to have transcripts of their interviews returned to them. No participants requested this, however.

All interviews were conducted in private rooms in the respective clinics in which only Author 1 and the study participant were present at a time convenient for the participants. This regarded both their general daily routine and individuals´ preferences regarding the time between the interview and the intake of their medication. Prior to the interviews, we assessed individuals’ level of intoxication, including their potential to provide informed consent and participate.

We are aware of debates on compensating participants in addiction research and agree with Souleymanov et al. (2016) on the importance of fair compensation with cash payments. Participants were informed of a compensation (20€ in cash) in the study information. Some likely participated due to the compensation, others had forgotten about it at the time of the interview and were surprised when Author 1 provided the compensation.

1.4 Interview guide:

Depending on participants´ individual circumstances (currently being in SIOT or in oral treatment, never or ever having been in SIOT), we developed seperate semi-structured interview guides. The interview guides were developed by Authors 1, 2, and 3 based on clinical experience and prior studies in health services research and were similar in their aim to answer the research questions:

-Why is SIOT is rarely used despite the benefits repeatedly shown in previous studies?

-What is the client perspective on

-barriers and enablers to initiation and maintenance of SIOT?

-challenges and potentials of the current form of treatment?

-concrete ways to improve SIOT?

The interview guides were not piloted but discussed in a focus group including people with lived experience in both oral treatment and SIOT. The focus group also included a person providing psychosocial support in the Berlin clinic (but not to the people living with OUD present during the focus group), and a trauma therapist who moderated the discussion. Author 1 was present to take notes during the focus group. After the first rounds of interviews, the researchers reflected upon the interview guides and felt like no changes were necessary.

Full interview guides (English version):

**For participants currently in SIOT**

What was your life like when you initiated SIOT?

Why did you decide to initiate SIOT?

What were your expectations, what were your goals?

Was there anything you worried about?

What is your experience with SIOT?

Do you think that it has helped you in any way?

If so, how? What has helped you the most?

If not, why not?

What do you like/dislike? What problems have you experienced?

How do you think SIOT could be improved?

Is there any additional support you would like to get but you are not receiving currently?

Did you ever think about ending the treatment?

If so, why? Can you tell me a bit more about that?

Is there anything else you would like to comment on that I have not asked you about?

**For participants currently in oral opioid substitution treatment who had in the past received SIOT**

What was your life like when you initiated SIOT?

Why did you decide to initiate SIOT?

What were your expectations, what were your goals?

Was there anything you worried about?

What was your experience with SIOT?

Do you think that it has helped you in any way?

If so, how?

If not, why not?

What did you like/dislike? What problems have you experienced?

How do you think SIOT could be improved? What do you think would have made your experience in SIOT better?

Can you tell me a bit more about the time you ended the treatment?

Would you consider going back into treatment?

If so, why?

If not, why not?

Is there anything else you would like to comment on that I have not asked you about?

**For participants currently in oral treatment who had never been in SIOT**

What was your life like when you initiated substitution treatment?

Why did you decide to initiate substitution treatment?

Are you aware of SIOT?

If so, what do you think of it?

Would you like to receive SIOT?

If so, why?

If not, why not?

Is there anything that would need to change for you to consider starting SIOT?

Is there anything else you would like to comment on that I have not asked you about?

2. Further information on data analysis

Data from all participant interviews were included in the analysis. A broad range of themes were derived from the data inductively. After familiarization with the material, Author 1 established categories reflective of participants´ perceptions of SIOT. These included both the dynamics underlying perceptions´ formation and the content of those perceptions. The emerging categories were continuously refined, and subcategories increasingly differentiated the category system. We report on the perceptions´ content elsewhere (Friedmann et al., 2023) and focus on their formation in the current article. In a cyclical-interactive approach, emerging ideas were put in context with prior analyses and repeatedly corroborated by referring back to participants´ quotes. Author 1 increasingly incorporated sociological literature into this process, which eventually led to the theoretical framework guiding the current article. Cross-case coding and the theoretical considerations for the analysis presented in this paper were paralleled by writing summaries on the formation and change of individual participants´ schemas of SIOT. This was done to complement patterns across interviews with individual accounts and identify potential contradictions. Our complete category system can be provided upon request from the corresponding author. This is the part of the category system used for the current article:


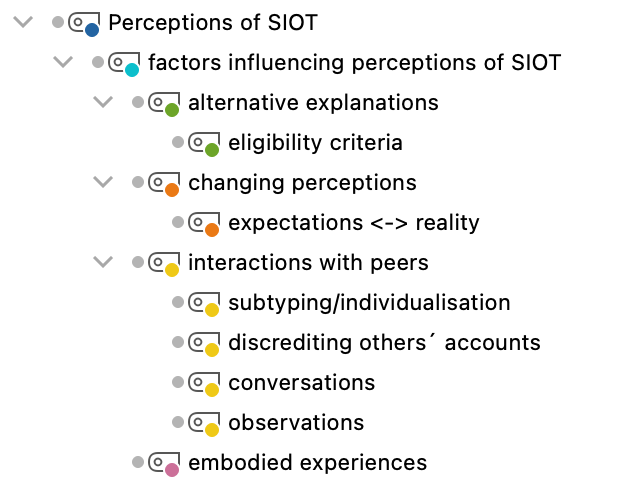


Category system as exported from MaxQDA (2022) software

Author 1 presented the category system and analytical approaches at interdisciplinary interpretation groups, colloquia, and congresses. These included

-Kolloquium “Versorgungsforschung” (colloquium on health services research, epidemiology, and public health) at Medizinische Hochschule Brandenburg

- Kolloquium der Nachwuchsgruppen von DGSMP und DGMS 2023 (colloquium for early career researchers by the German association on medical sociology and the German association on social medicine and prevention)

-Forschungswerkstatt “Allgemeinmedizin” (interpretation group on general and social medicine and public health) at Charité Berlin

-Forschungswerkstatt “Qualitative Methoden” (interpretation group on qualitative research methods in public health and implementation science) at Zentrum für öffentliches Gesundheitswesen und Versorgungsforschung Tübingen

-31. Kongress der deutschen Gesellschaft für Suchtmedizin (31st congress of the German society for addiction medicine)

**References**

Friedmann, Z., Binder, A., Kinkel, H.-T., Kühner, C., Zsolnai, A., & Mick, I. (2023). Exploring Patients’ Perceptions on Injectable Opioid Agonist Treatment: Influences on Treatment Initiation and Implications for Practice. European Addiction Research, 1-11. https://doi.org/10.1159/000535416

Malterud, K., Siersma, V.D., & Guassora, A.D. (2016). Sample Size in Qualitative Interview Studies: Guided by Information Power. Qual Health Res, 26, 1753-1760.

Souleymanov, R., Kuzmanović, D., Marshall, Z., Scheim, A.I., Mikiki, M., Worthington, C., et al. (2016). The ethics of community-based research with people who use drugs: results of a scoping review. BMC Medical Ethics, 17, 25.
